# Flexural Behavior of Innovative Glass Fiber-Reinforced Composite Beams Reinforced with Gypsum-Based Composites

**DOI:** 10.3390/polym16233327

**Published:** 2024-11-27

**Authors:** Yiwen Liu, Bo Su, Tianyu Zhang

**Affiliations:** Faculty of Civil Engineering and Mechanics, Jiangsu University, Zhenjiang 212000, China; liuyiwen0426@163.com (Y.L.); zty9152024@163.com (T.Z.)

**Keywords:** gypsum, polyvinyl acetate fibers, GFRP, composite beams, coagulation characteristics

## Abstract

Glass Fiber-Reinforced Composite (GFRP) has found widespread use in engineering structures due to its lightweight construction, high strength, and design flexibility. However, pure GFRP beams exhibit weaknesses in terms of stiffness, stability, and local compressive strength, which compromise their bending properties. In addressing these limitations, this study introduces innovative square GFRP beams infused with gypsum-based composites (GBIGCs). Comprehensive experiments and theoretical analyses have been conducted to explore their manufacturing process and bending characteristics. Initially, four types of GBIGC—namely, hollow GFRP beams, pure gypsum, steel-reinforced gypsum, and fiber-mixed gypsum-infused beams—were designed and fabricated for comparative analysis. Material tests were conducted to assess the coagulation characteristics of gypsum and its mechanical performance influenced by polyvinyl acetate fibers (PVAs). Subsequently, eight GFRP square beams (length: 1.5 m, section size: 150 mm × 150 mm) infused with different gypsum-based composites underwent four-point bending tests to determine their ultimate bending capacity and deflection patterns. The findings revealed that a 0.12% dosage of protein retarder effectively extends the coagulation time of gypsum, making it suitable for specimen preparation, with initial and final setting times of 113 min and 135 min, respectively. The ultimate bending load of PVA-mixed gypsum-infused GFRP beams is 203.84% higher than that of hollow beams, followed by pure gypsum and steel-reinforced gypsum, with increased values of 136.97% and 186.91%, respectively. The ultimate load values from the theoretical and experimental results showed good agreement, with an error within 7.68%. These three types of GBIGCs with significantly enhanced flexural performance can be filled with different materials to meet specific load-bearing requirements for various scenarios. Their improved flexural strength and lightweight characteristics make GBIGCs well suited for applications such as repairing roof beams, light prefabricated frames, coastal and offshore buildings.

## 1. Introduction

Glass Fiber-Reinforced Composite (GFRP) is a material composed of resin and glass fiber, which is valued in the construction industry for its high strength and cost-effectiveness. The decreasing cost of raw materials and advancements in composite manufacturing technologies, such as pultrusion or vacuum introduction, have contributed to the growing popularity of GFRP in civil engineering [1,2,3]. Noteworthy applications of GFRP in civil engineering include the use of GFRP sandwich panels for bridge decks [4], lightweight slabs for the restoration of wooden or masonry buildings [5,6], composite crashworthy devices [7], ship deck panels [8], emergency housing, and various new construction projects [9]. However, it is important to note that GFRP, being inherently brittle, exhibits weaknesses in terms of stiffness, stability, and durability [10,11,12]. Many researchers are actively exploring ways to enhance its structural and mechanical performance [13,14].

An effective strategy for enhancing the mechanical performance of GFRP involves combining it with concrete to create composite structures. Robinson et al. [15] conducted a study on the bonding effects of short-span rectangular GFRP beams filled with concrete. The results demonstrated that the strength and stiffness of the concrete-filled GFRP beams were twice as high as those of unbonded GFRP beams. In a similar vein, Ahmed Abouzied et al. [16] investigated the flexural behavior of rectangular FRP beams filled with reinforced concrete. Their findings revealed that the strength of concrete and the thickness of GFRP beams were the primary factors influencing the flexural performance of infilled FRP beams. Additionally, Ahmed et al. [17] and Majid Muttashar et al. [18] highlighted improvements in the ultimate bearing capacity, ductility, and energy absorption of concrete-infilled GFRP beams with an increase in FRP beam thickness and concrete compressive strength. It is worth noting, however, that the incorporation of concrete significantly increases the weight of the structure, which may have detrimental effects on its overall performance.

In recent research efforts, there has been a focus on using lighter infilling materials instead of concrete to reduce structural weight. For instance, Huang Li et al. [19] introduced three types of wood–GFRP beams (plate form, slot form, and inverted T-shaped form). Their study revealed that slot GFRP–wood beams exhibited the best mechanical properties, showing a 26.43% increase in the ultimate load-carrying capability compared to that of the reference GFRP beams. C. Yoganantham et al. [20] conducted research on the flexural performance of pultruded glass fiber-reinforced polymer (GFRP) beams filled with a large volume of fly ash engineering cementitious composite (HVFAECC). The results indicated that the bearing capacity of tension GFRP beams filled with HVFAECC was significantly higher than that of hollow GFRP beams. Fubin Zhang et al. [21] proposed a novel composite sandwich structure that incorporates GFRP skins, cold-formed profiled steel plate, and lightweight balsa wood. The test findings demonstrated that the ultimate bearing capacity of the composite structure increased by 68–194% compared to that of hollow structures. These studies highlight the potential of using alternative, lighter materials to enhance the mechanical properties of GFRP structures while addressing concerns related to structural weight.

Gypsum, which is recognized as a cementing material, finds widespread use in the construction industry due to its excellent features such as its light weight, fire resistance, and fast setting time. Gypsum can be sourced from natural reserves or industrial by-products, and thus presents an effective means of waste utilization. The properties of fiber-reinforced materials have become increasingly common in recent years. Palomo IRI et al. [22] highlight the significant potential of utilizing ultra-high-performance fibers to enhance the mechanical properties of concrete, thereby improving the structural performance of reinforced concrete (RC) beam–column joints. In efforts to enhance gypsum’s physical and mechanical properties, researchers have conducted experiments in which they have added various fibers to gypsum [23,24]. These fibers include paper, polystyrene, polyester, seagrass [25,26], and recycled waste gypsum [27,28,29,30,31]. Cong Zhu et al. [32] investigated the physical and mechanical properties of polyvinyl alcohol and polypropylene fiber-reinforced gypsum-based composites. Their results demonstrated a significant improvement in the flexural strength of hardened gypsum-based composites with the addition of polyvinyl alcohol fiber. Aldi Kuqo et al. [33] studied the mechanical properties of two natural fibers: Mediterranean seaweed and pine gypsum. They discovered that seaweed fiber and wood fiber could enhance crucial properties of gypsum, including its compressive strength, impact resistance, and hardness. Zhenxing Li et al. [34] explored the effects of basalt fiber, glass fiber, and polyvinyl alcohol fiber on the setting time, extension diameter, water absorption, and bending strength of fiber-reinforced gypsum-based composites. Their contributions have established the positive impact of fibers on the physical and mechanical properties of gypsum composites. However, there remains a gap in research concerning the flexural behavior of gypsum-based composite structures.

In summary, while load-bearing GFRP pipes can meet the necessary weight requirements, they still exhibit certain limitations in terms of their flexural and compressive performance compared to traditional reinforced concrete beams. Focusing on both lightweight characteristics and sufficient strength, this study selected gypsum as the matrix material and incorporated reinforcement and PVA to address the brittleness of the structure. This enhanced the ultimate load-bearing capacity while introducing certain plasticity characteristics, thereby meeting the requirements of practical engineering applications. For example, this composite material system could be widely applied in structural elements such as beams and columns in factory buildings, emergency structures, prefabricated buildings, and other construction scenarios that demand both light weight and a high load-bearing capacity.

This research focuses on three types of gypsum-based composite GFRP beams: pure gypsum, steel-reinforced gypsum, and fiber-reinforced gypsum composites. The primary focus of the investigation lies in the bending properties of these composite beams. Section 2 provides a detailed description of the material preparation process, including tests to evaluate the setting characteristics of gypsum and the influence of polyvinyl acetate (PVA) on the mechanical properties of gypsum blocks. Additionally, comprehensive details of the four-point bending test procedure are provided. Section 3 presents the theoretical calculation formulas for the load-bearing capacity of four types of components. Subsequently, Section 4 analyzes the results of the four-point bending experiments, offering a detailed examination of the failure modes observed during the experiments, and compares the theoretical and experimental results. Section 5 outlines the innovations of this study and summarizes the bending behavior exhibited by square GFRP beams filled with gypsum-based composites.

## 2. Materials and Methodology

The main materials used for the fabrication of GBIGCs include GFRP, gypsum, PVA, and steel bars.

### 2.1. Pultruded GFRP Beams’ Properties

**Figure 1:**
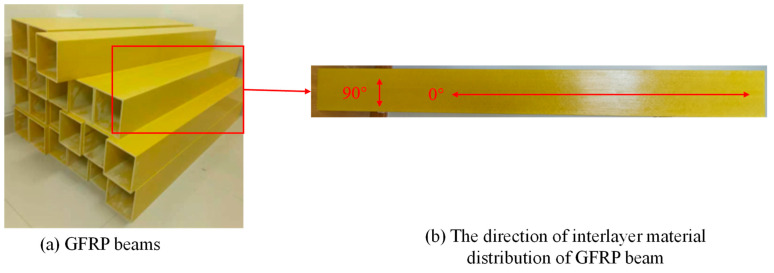
GFRP beams and directions.

**Figure 2:**
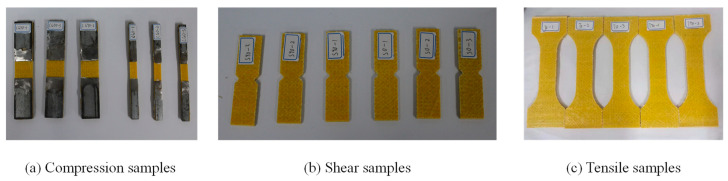
Samples in two directions of GFRP.

As shown in Figure 1a, the hollow pultruded GFRP square beams (length: 1.5 m, section area: 150 mm × 150 mm) used in this study were manufactured by Si Tong FRP Co., Ltd. (Soochow, China). In the pultrusion process, the material matrix is a thermosetting resin, and the fibers used are glass fibers. Through traction pultrusion and heating, the material is cured and formed into a single unit without differences in interlayer properties in the thickness direction. However, fibers are typically aligned along the axial direction of the profile. This fiber arrangement results in high strength and stiffness in the axial direction, allowing the profile to effectively withstand tensile, compressive, and bending mechanical stresses [35,36,37]. In this study, the GFRP material adopted this pultrusion process, which demonstrated the differences in mechanical properties in different directions. Therefore, the mechanical properties test was carried out in two directions of 0° and 90°, and the tensile, compression, and shear properties were tested, respectively. The direction is shown in Figure 1b. The direction of the extended square tube was set to 0°, and the direction perpendicular to 0° was 90°. The test samples are shown in Figure 2. The testing standards utilized were as follows: the in-plane shear performance testing standard was ASTM D5379-19; the in-plane tensile performance testing standard was ASTM D3039-07, and the in-plane compression performance testing standard was ASTM D3410-16 [38,39,40]. The three performance tests easily demonstrated the characteristics of orthotropic stratification, and the elastic modulus in the 0° direction was higher than that in the 90° direction, as shown in Table 1.

### 2.2. Desulfurization Gypsum

The gypsum was desulfurized and provided by Shandong Dong Taiyuan Gypsum Technology Co. Ltd. (Zaozhuang, China), and its physical properties are shown in Table 2.

### 2.3. PVA 

The PVA was provided by Shanghai Chenqi Chemical Technology Co., Ltd. (Shanghai, China) and was used to improve gypsum’s performance in light of its high bonding strength, corrosion resistance, and hydrophilicity. The physical parameters and morphology of PVA are shown in Table 3.

### 2.4. Steel Bars

Hot rolled steel bars of grade HRB400 [41] were used for the fabrication of steel-reinforced gypsum beams; these were provided by Kunshan Guibang Steel Co., Ltd. (Kunshan, China). The diameters of the longitudinal reinforcement and stirrup were 8 mm and 6 mm, respectively. The basic mechanical performance of the steel bars is listed in Table 4.

### 2.5. Coagulation Test

**Figure 3:**
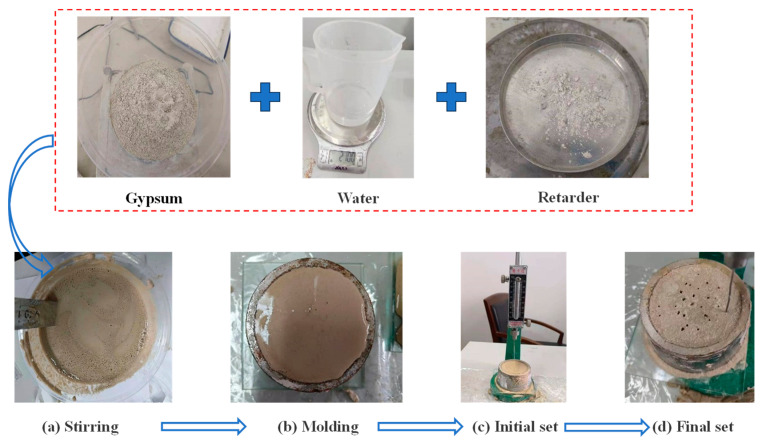
Gypsum coagulation tests.

The initial and final setting times of pure gypsum are 5 min and 10 min, respectively; these times were insufficient for the pouring process. Therefore, a protein retarder (manufactured by Beijing Long Tengda Chemical Company) was introduced to extend the setting time, and coagulation tests were conducted to assess the retarder’s impact on the condensation time. The key steps of the main test, as illustrated in Figure 3, are as follows:

(a) Weigh 300 g of gypsum, 210 g of water, and the specified ratio of retarder. Thoroughly mix the gypsum and retarder, and within 5 s, pour the sample into water to achieve a uniform slurry, as shown in Figure 3a.
(b) Pour the evenly stirred slurry into the ring mold, ensuring the slurry flushes to the upper end of the ring mold, as depicted in Figure 3b.
(c) Record the initial setting time, defined as the period from the contact of the sample with water to the first instance when the steel needle cannot touch the glass-bottom plate, as demonstrated in Figure 3c.
(d) Record the final setting time, defined as the period from the contact of the sample with water to the time when the depth of the steel needle inserted into the slurry is below 1 mm for the first time, as shown in Figure 3d.

### 2.6. Fabrication of Gypsum Blocks for Mechanical Test

**Figure 4:**
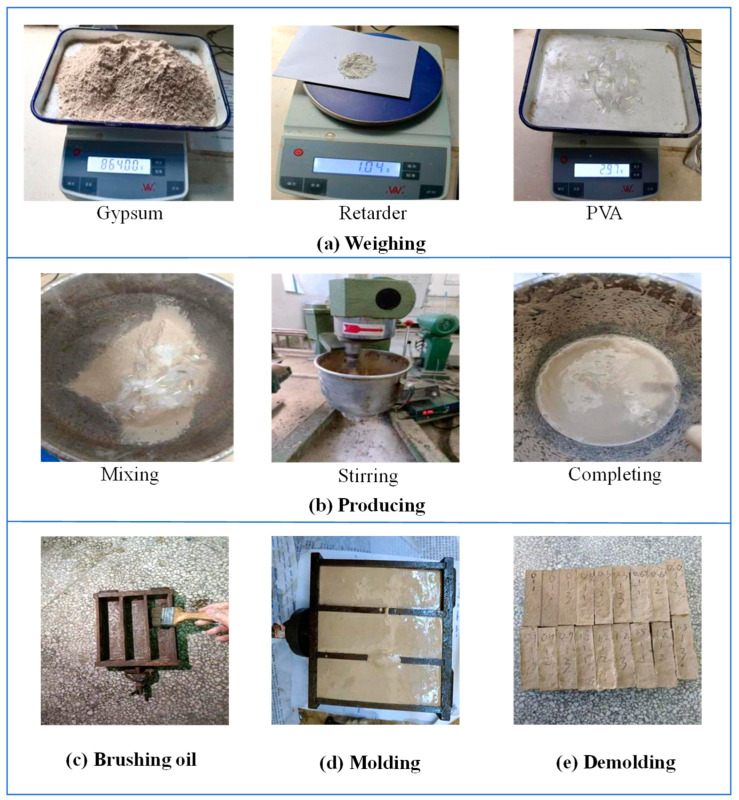
Manufacturing process of PVA gypsum blocks.

As depicted in Figure 4, PVA gypsum test blocks were fabricated in the laboratory, incorporating varying dosages (0~1.5%) of PVAs. The primary steps involved in this process are as follows:

(a) Weighing: Measure 864 g of gypsum, 605 g of water, 1.04 g of retarder, and PVAs with dosages ranging from 0% to 1.5%.
(b) Mixing and stirring: Pour the aforementioned materials into the blender and stir until a homogeneous mixture is achieved.
(c) Brush oil onto the inner surfaces of the molds.
(d) Pour the mixture into the molds, allowing the blocks to take shape over the course of a day.
(e) Demold the blocks and cure them in a curing box for a duration of 3 days.

The gypsum blocks were subjected to flexural and compressive tests according to the standard GB/T50081-2019 [42], and we evaluated how the dosage of PVAs affected its mechanical performance.

### 2.7. Four-Point Bending Tests

As outlined in Table 5, four types of GFRP square beams were designed, comprising the hollow GFRP beam (H-G beam), pure gypsum GFRP beam (G-G beam), reinforced gypsum GFRP beam (R-G beam), and PVA gypsum GFRP beam (P-G beam). Two identical test specimens were created for each type, resulting in a total of eight GFRP square beams that underwent four-point bending tests.

The material properties of the gypsum, retarder, and PVAs are as described in Section 2.1.

**Figure 5:**
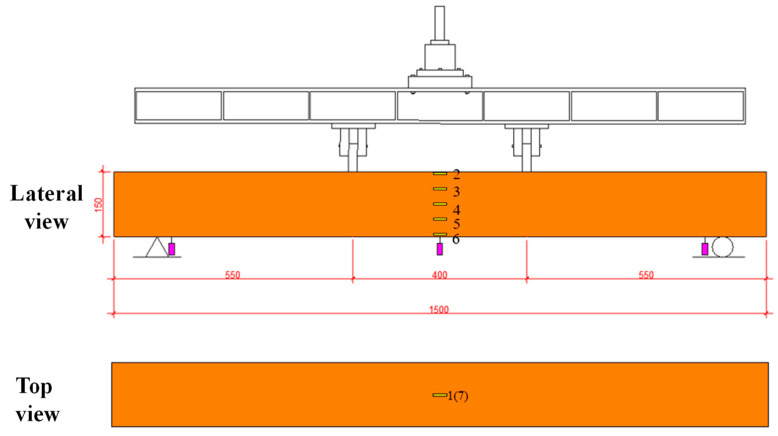
The 50 T self-balancing device and layout of sensors.

The four-point bending tests were conducted using the 50 T self-balancing device at Jiangsu University (ZF-F500). In preparation, seven strain gauges and three dial indicators were strategically installed, as shown in Figure 5. Among the seven strain gauges, five (No. 2~No. 6) are evenly distributed along the beam web, while two (No. 1 and No. 7) are positioned at the midpoint of the upper and lower panels. The three dial indicators were employed to record deflection, with two placed at the bottom ends and one situated at the bottom midpoint. The test standard is GB/T 1449 [43].

The primary test procedures are as follows:

(a) Prepare square GFRP beams filled with gypsum-based composites and affix the strain gauges using silicone rubber.
(b) Install the GFRP beams in the vertical loading reaction system device, adjusting the position of supports and their height.
(c) Connect the static resistance strain gauges with wires to form a quarter bridge and install displacement indicators.
(d) Preload the specimen with 2.5 kN to ensure full contact between the loading device and the test beam, then unload it back to zero.
(e) Apply a continuous load on the specimen with loading steps of 2.5 kN until failure occurs. Record load levels, strain, and displacement values throughout the entire loading process.

## 3. Theoretical Study

The bending and shearing deformation value of GBIGCs can be calculated out according to Equations (1) and (2), respectively [2,15].

(1) $$\delta_{b}=\frac{Pa}{48EI}(3l^{2}-4a^{2})$$

(2) $$\delta_{s}=\frac{Pa}{GA}f_{s}$$

By adding Equations (1) and (2), the deflection of GBIGCs can be written as shown in Equation (3) [2,15].

(3) $$\delta =\delta_{b}+\delta_{s}=\frac{Pa}{48EI}(3l^{2}-4a^{2})+\frac{Pa}{GA}f_{s}$$

where δ is the deflection of the composite beam; $\delta_{b}$ is the bending deflection; $\delta_{s}$ is the shear deflection; P is the maximum load; E is the elastic modulus; I is the moment of inertia of the section; a is the shear span; l is the calculation span of the simply supported beam; G is the shear modulus; A is the section area; and $f_{s}$ is the shape coefficient of cross-section shear stress.

The values of EI, GA and $f_{s}$ can be derived by Equations (4)–(6) [14,44].

(4) $$EI=\sum E_{i}I_{i}$$

(5) $$GA=\sum G_{i}A_{i}$$

(6) $$f_{s}=\frac{A}{I^{2}}\int_{A}\frac{S_{i}^{2}}{{b_{i}}^{2}}d_{A}$$

where $E_{i}$ is the elastic modulus of each component; $I_{i}$ is the moment of inertia of each component; $G_{i}$ is the shear modulus of each component; $A_{i}$ is the area of each component; $S_{i}$ is the first moment of the area of each component; and $b_{i}$ is the width of each component of the composite beam.

According to the above Equations (3)–(6), the ultimate load value of GFRP square beams can be deduced by Equation (7).


(7) $$P=\frac{\delta }{\frac{a(3l^{2}-4a^{2})}{48EI}+\frac{a}{GA}f_{s}}$$

## 4. Results and Discussion

### 4.1. Coagulation Test Results

**Figure 6:**
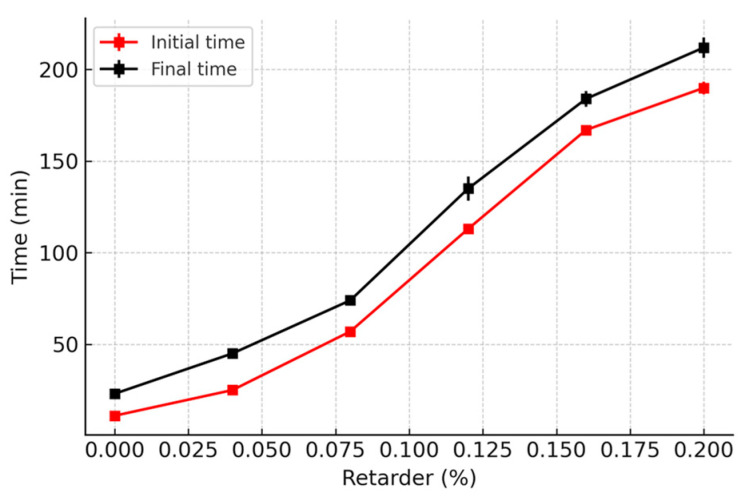
Relation between the retarder and the setting time of gypsum.

The relationship between the protein retarder dosage and the gypsum setting time is illustrated in Figure 6. The graph indicates that as the retarder dosage increases, both the initial and final setting times of gypsum extend. In the subsequent experiments, a protein retarder dosage of 0.12% is employed for gypsum or gypsum-based composites. The initial and final setting times are recorded as 113 min and 135 min, respectively, and these times are deemed suitable for specimen preparation.

### 4.2. Results of the Flexural and Compressive Strength Tests on Gypsum Blocks 

**Figure 7:**
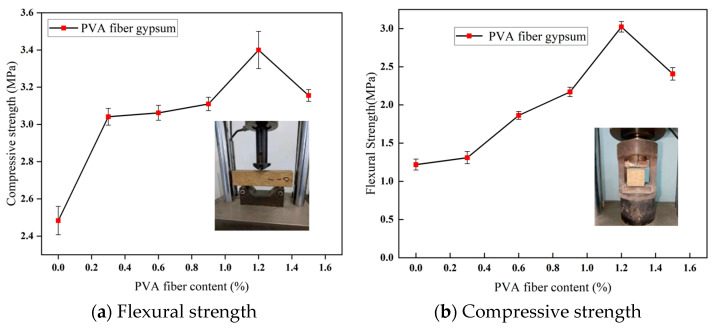
Mechanical performance of PVA gypsum blocks.

The test results are presented in Figure 7. As depicted in the figure, with the increase in PVA fiber content (0% to 1.2%), the flexural and compressive strength of PVA gypsum material increase, reaching maximum values of 2.97 MPa and 3.4 MPa, respectively, at a PVA dosage of 1.2%. However, the strength exhibits a downward trend when the dosage of PVA increases to 1.5%. Therefore, a PVA dosage of 1.2% is selected for the subsequent tests. When the PVA content increases from 0% to 1.2%, both flexural strength and compressive strength significantly improve. This is primarily due to the fiber’s crack-bridging effect, which enhances the material’s toughness and slows the propagation of microcracks, thus improving its load-bearing capacity. However, when the fiber content increases to 1.5%, the strength begins to decrease. This may be attributed to fiber agglomeration leading to uneven distribution and causing stress concentration. Moreover, an excess of fibers could disrupt the continuity of the gypsum matrix, reducing the effective bonding area of the matrix. Additionally, the bond strength at the fiber matrix interface may weaken, leading to increased interface debonding. These results suggest that the optimal fiber content is 1.2%; at this percentage, a balance between enhanced performance and material uniformity is achieved, maximizing the material’s overall properties.

### 4.3. Results of the Four-Point Bending Tests 

**Figure 8:**
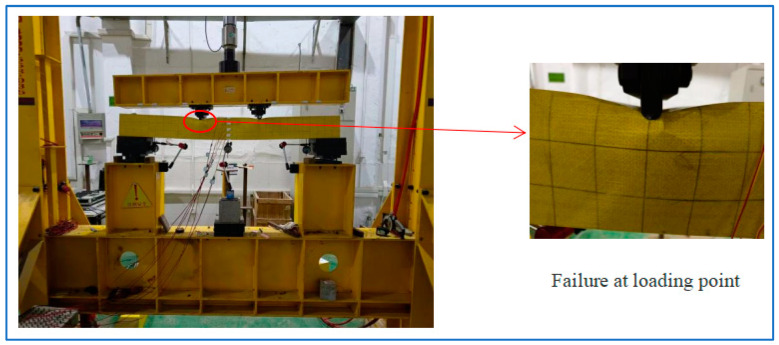
Loading of GFRP beams.

As illustrated in Figure 8, the hollow GFRP specimens undergo loading from two symmetric concentrated loads. As the loading level increases, the specimens generate a more pronounced sound, indicating deeper fiber fractures. Ultimately, all the specimens lose their strength due to the crushing failure of the GFRP panel at the loading points. Moreover, as shown in Figure 8, localized fiber damage leads to fracture failure, with a simultaneous loss of local load-bearing capacity, accompanied by noticeable buckling on the sides. This results in overall instability of the component. Therefore, to prevent buckling, the study introduced a series of filler materials, such as the gypsum matrix, to mitigate this issue.

**Figure 9:**
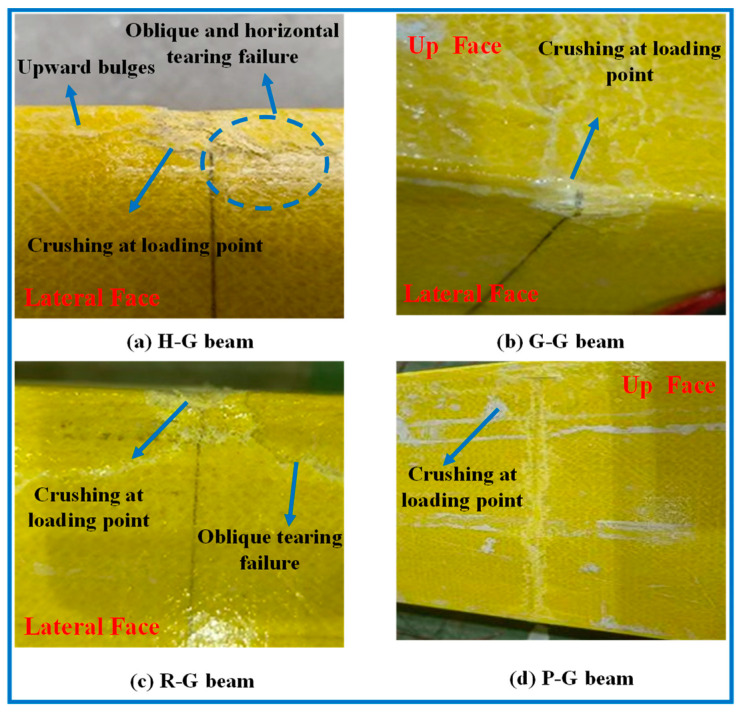
Local failure pattern at loading points of hollow GFRP or GBIGC specimens.

The hollow GFRP or GBIGC specimens exhibit different failure patterns around the loading points. In the case of the H-G beam shown in Figure 9a, the GFPR panel experiences intensive crushing at the loading points, especially at the junction between the upper flange and the web. This induces oblique and horizontal tearing failure, accompanied by an obvious upward bulge near the loading points. For the G-G beam in Figure 9b, it also displays intensive crushing at the loading points, but without oblique or horizontal tearing failure. This may be due to the good compatibility between the two materials in the G-G beam. The gypsum matrix provides supporting strength, which helps to avoid an excessive concentration of tensile stress to some extent. When the internal gypsum undergoes damage, it leads to localized crushing, while the tearing failure is mitigated. In the case of the R-G beam in Figure 9c, it not only shows intensive crushing at the loading points but also exhibits obvious oblique tearing failure below the crushing zone. When initial cracks occur locally, both the gypsum and the reinforcement can still function effectively. As the load increases, the gypsum gradually crushes and loses its effectiveness, but since the reinforcement remains intact, the material, primarily composed of GFRP, still has residual stress. The overall structure retains load-bearing capacity, which leads to further damage after GFRP failure. Compared to other structures, localized fractures in this case are more pronounced. However, for the P-G beam in Figure 9d, it displays relatively light local compression failure at the loading points, indicating better local compressive mechanical performance. Compared to ordinary gypsum, PVA-reinforced gypsum exhibits a significant improvement in both compressive and flexural strength. Additionally, the gypsum matrix shows good compatibility with GFRP, which effectively enhances the stiffness and strength at the loading points, reducing the occurrence of concentrated stress.

#### 4.3.1. Ultimate Limit State Analysis

**Figure 10:**
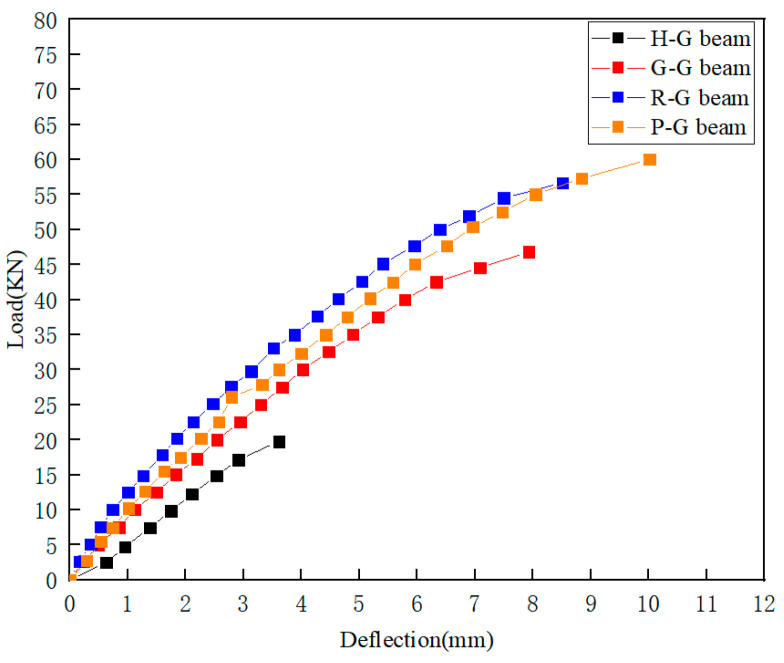
Mid-span deflection of test beams.

The load–deflection curves of the GFRP square beams are presented in Figure 10. The curves illustrate that the gradient of the curve, which represents rigidity, for G-G beam, R-G beam, and P-G beam is greater than that of H-G beam. R-G beam exhibits the greatest stiffness, followed by P-G beam and G-G beam. The ultimate load values of the infilled beams are significantly improved compared to that of H-G beam. It was also found that the stiffness of the R-G beam is the highest, but its ultimate strength is 7% lower than that of the P-G beam. This is because the strength of the reinforcement itself is higher than that of the PVAs, which results in higher stiffness at the initial stage of the experiment. However, as the load increases, the bond between the gypsum and reinforcement in the R-G beam is not optimal, leading to a failure similar to over-reinforcement, where the bending resistance of the materials prevents them from working together effectively. In contrast, the fibers and gypsum in the P-G beam show good compatibility, with the fibers having strong ductility. This results in higher load-bearing capacity and greater displacement at the monitoring points, making the P-G beam more suitable for practical weight-reduction applications.

The test results for the ultimate load values and their corresponding deflections are summarized in Table 6. The ultimate loads for H-G, G-G, R-G, and P-G beams are 19.759 kN, 46.822 kN, 56.690 kN, and 60.035 kN, respectively. The maximum displacements occur at the mid-span, measuring 3.610 mm, 7.928 mm, 8.513 mm, and 10.013 mm, respectively. Consequently, the ultimate loads of G-G, R-G, and P-G beams are increased by 136.97%, 186.91%, and 203.84%, respectively, compared with that of the H-G beam. The increase in ultimate load is attributed to the synergistic effect between the gypsum matrix and the GFRP interface. Additionally, after gypsum filling, its compressive strength provides strong support for the GFRP, leading to stress redistribution on the GFRP’s surface. This enhances the local compressive capacity and increases the overall load-bearing capacity. The reinforcement and PVAs, with their high tensile strength, further enhance the overall strength by combining with the compressive performance of the gypsum.

#### 4.3.2. Strain Distribution Analysis

**Figure 11:**
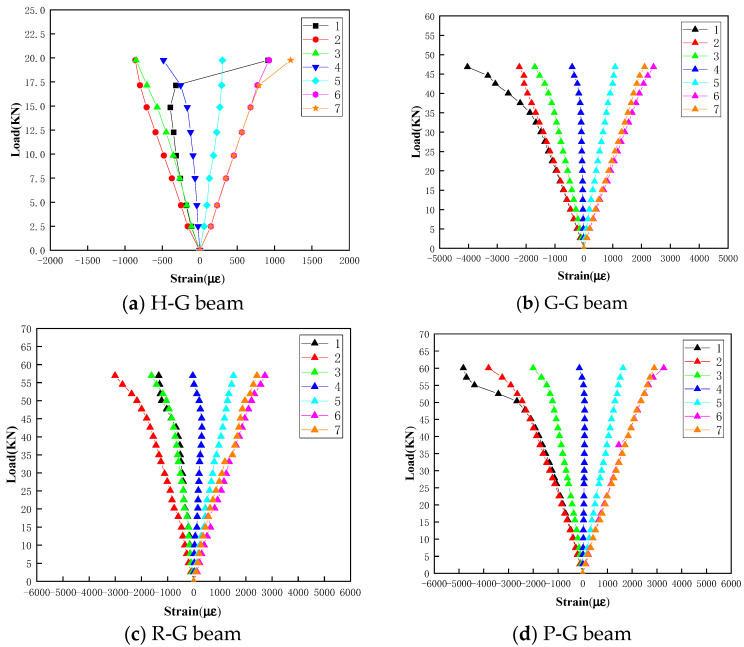
Load–strain curves of different gauges.

The load–strain curves of the four types of specimens are drawn in Figure 11. The strain values of No.1~No.3 gauges are negative values, as they are installed in the compression zone, and the strain values of No.5~No.7 gauges are positive values because they are installed in the tensile zone. The strain value of No.4 gauge is near zero, as it is located at a neutral axis. Generally, the absolute strain values at all gauges of G-G, R-G and P-G beams increase with the loading level, which demonstrates the linear load–strain relationship.

As shown in Figure 11a, for the No.1 gauge of the H-G beam, the strain increases with higher loading values and reaches its minimum negative value at a loading value of 14.4 kN. However, as the load level continues to increase, the absolute value of the strain decreases and suddenly jumps to a larger positive value (tensile strain) when the loading value reaches 19.759 kN. This behavior is attributed to local buckling and the upward bulge of the GFRP upper surface around the No.1 gauge near the failure stage, which induces tensile strain. For the G-G, R-G, and P-G beams, there is no such strain jump phenomenon, as shown in Figure 11c,d.

### 4.4. Discussion

#### 4.4.1. Height-Wise Strain Distribution Analysis

**Figure 12:**
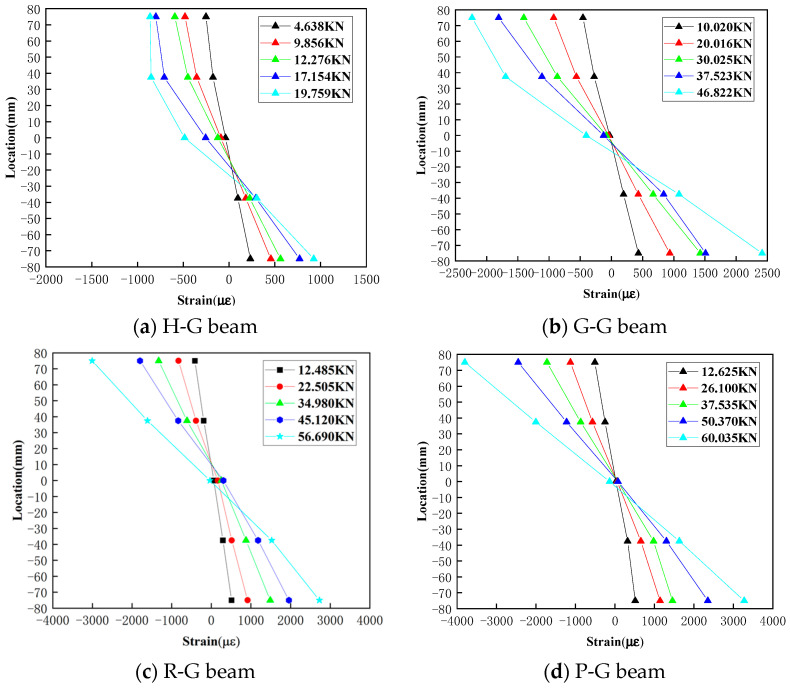
Strain distribution along web height.

Based on the strain data from gauges (No.2~No.5) installed on the webs of the specimens, strain distribution curves along the height can be derived, as depicted in Figure 12. Generally, the strain along the web height conforms to a linear distribution rule, aligning with the plane section assumption of beam deformation in material mechanics [40]. In Figure 12a, it can be observed that the curves at 4.638 kN and 9.856 kN are linear, indicating that the material is still in the elastic phase. As the applied load increases, the curves at 12.276 kN, 17.154 kN, and 19.759 kN exhibit nonlinear characteristics, with some regions entering the plastic phase. At higher load levels, strain changes rapidly at the top, indicating that yielding has occurred.

In Figure 12b, only the 46.822 kN curve gradually shows nonlinear characteristics, but compared to the H-G beam, the strain range is larger. This suggests that using gypsum as the matrix material effectively improves overall integrity, reducing the impact of localized damage on the overall strength.

Figure 12c,d show that the ultimate strain can reach 3000 and 3400, respectively, which is approximately 200% higher than the strain of 1000 observed in the hollow pipe, demonstrating a significant improvement. Additionally, it can be seen that the PVA gypsum matrix beams have a larger strain range, indicating that the tensile performance provided by the PVAs in the gypsum matrix has been fully utilized.

#### 4.4.2. Theoretical Analysis

The theoretical calculation results of the ultimate load values are provided in Table 7, and experimental results are also included for comparison. As shown in the table, the theoretical ultimate load values for H-G, G-G, R-G, and P-G beams are 19.983 kN, 50.428 kN, 53.935 kN, and 65.027 kN, respectively. The relative errors between theoretical and experimental values are 1.12%, 7.15%, 5.11%, and 7.68%, respectively, indicating a good match between theoretical predictions and experimental results. The results presented by the four stress–strain curves are generally consistent with the assumption of plane sections, so the errors in the calculated results are all within 8%. The relatively low error for the H-G beam indicates that the theoretical model can effectively reflect the beam’s load-bearing behavior under the given conditions. However, due to the nonlinear characteristics of the gypsum matrix material, there is some error in the other three materials. The error in the P-G beam is also closely related to the distribution pattern of the PVAs within the gypsum. Nevertheless, the overall error for all three materials is within an acceptable range, and this calculation theory generally meets engineering calculation requirements.

## 5. Conclusions

The bending properties of novel GFRP square beams infilled with gypsum-based composites (GBIGCs) were comprehensively studied through experimental and theoretical analyses. The main conclusions are summarized as follows:

- Protein retarder sensitivity: The setting time of gypsum was found to be sensitive to protein retarder. An optimal dosage of 0.12% protein retarder effectively prolongs the initial and final setting time from 5 min and 10 min to 113 min and 135 min, respectively, making it suitable for specimen preparation.
- PVA composite gypsum strength: The flexural and compressive test results of gypsum blocks indicate that as the PVA content increases from 0% to 1.2%, the strength of PVA gypsum continuously increases. However, a downward trend is observed as the PVA dosage changes from 1.2% to 1.5%. The optimal dosage of PVA is determined to be 1.2%, enhancing the flexural and compressive strength of PVA composite gypsum from 1.17 MPa and 2.50 MPa to 2.97 MPa and 3.4 MPa, respectively.
- Ultimate load values of GRFP square beams: In the four-point bending tests, the ultimate load values of pure gypsum, reinforced gypsum, and PVA gypsum infilled GFRP square beams are 46.822 kN, 56.690 kN, and 60.035 kN, respectively. Compared with the ultimate load value of hollow GFRP square beams (19.759 kN), the ultimate bending capacity of GFRP square beams infilled with gypsum, reinforced gypsum, and PVA gypsum increases by 136.97%, 186.91%, and 203.84%, respectively. Among them, PVA gypsum GFRP square beams exhibit the best mechanical performance.
- Strain distribution and theoretical equations: The strain distribution along the height at the middle span section generally meets the plane section assumption under different load levels. Theoretical equations are established to calculate the ultimate load values, whose results align well with experimental values.

The beam dimensions studied in this paper have certain limitations, as the beam length is 1500 mm, while in actual building structures, beam lengths typically exceed 2 m. The mechanical performance of such beam structures, particularly when they have varying cross-sections and span large distances, requires further investigation. Secondly, the study of the microscopic mechanical properties is somewhat limited. Specifically, how the internal reinforcement behaves under mechanical action and failure during four-point bending with gypsum-based filling materials, as well as the distribution characteristics of PVAs, are key areas for future research. Finally, the response of GBIGCs under different load conditions, such as uniformly distributed loads, dynamic impacts, and seismic performance, needs to be further studied and addressed for practical applications.

## Figures and Tables

**Table 1:** Tensile properties of GFRP.

Direction	Tensile Strength/MPa	Modulus of Elasticity/GPa	Compressive Strength/MPa	Modulus of Elasticity/GPa	Shear Strength/MPa	Modulus of Elasticity/GPa
0°	321.7	22.00	266.7	21.28	71.7	9.87
90°	28.8	2.65	57.8	4.45	52.4	9.76

**Table 2:** Physical parameters of gypsum.

Material	Water Requirement for Normal Consistency	Setting Times		Powdery Degree/200 Mesh	Whiteness/%
		Initial	Final		
Desulfurized gypsum	70%	5 min	10 min	0.01	50

**Table 3:** Physical parameters and morphology of PVA.

Name	Diameter/ μm	Length/mm	Density/ g/cm^3^	Tensile Strength/MPa	Modulus of Elasticity/GPa	Elongation at Break
PVA	15.09	12	1.29	1830	40	6.9%

**Table 4:** Mechanical parameters of Rebars.

Grade	Modulus of Elasticity/MPa	Poisson’s Ratio	Yield Stress/MPa
HRB400 steel bars	200,000	0.3	400

**Table 5:** Four kinds of GFRP square beams.

Types	Length/mm	Width/mm	Height/mm	End Face Style
K-G beam	1500	150	150	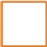
S-G beam	1500	150	150	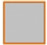
G-G beam	1500	150	150	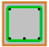
P-G beam	1500	150	150	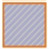

**Table 6:** Test results of H-G and GBIGC specimens.

Sample	Ultimate Load/kN	Average Value/kN	Mid-Span Deflection/mm	Average Value/mm
H-G-1	19.537	19.759	3.51	3.610
H-G-2	19.980		3.71	
G-G-1	45.130	46.822	7.62	7.928
G-G-2	48.514		8.235	
R-G-1	53.880	56.690	8.635	8.513
R-G-2	59.500		8.39	
P-G-1	60.070	60.035	9.495	10.013
P-G-2	60.000		10.53	

Note: Each type of GFRP square beam has two specimens, represented by the number 1 or 2.

**Table 7:** Theoretical calculation.

Type	f/mm	Experimental Ultimate Load $P_{u}$/kN	Theoretical Ultimate Load $P_{ul}$/kN	Relative Error
H-G beam	3.610	19.759	19.983	1.12%
G-G beam	7.928	46.822	50.428	7.15%
R-G beam	8.513	56.690	53.935	5.11%
P-G beam	10.013	60.035	65.027	7.68%

## Data Availability

The raw data supporting the conclusions of this article will be made available by the authors on request.

## References

[1] Alnahhal W., Aref A. (2008). Structural performance of hybrid fiber reinforced polymer–concrete bridge superstructure systems. Compos. Struct..

[2] Chakrabortty A., Khennane A., Kayali O., Morozov E. (2011). Performance of outside filament-wound hybrid FRP-concrete beams. Compos. Part B Eng..

[3] Hadi M.N., Wang W., Sheikh M.N. (2015). Axial compressive behaviour of GFRP beams reinforced concrete columns. Constr. Build. Mater..

[4] Keller T., Schollmayer M. (2004). Plate bending behavior of a pultruded GFRP bridge deck system. Compos. Struct..

[5] Evernden M.C., Mottram J.T. (2012). A case for houses to be constructed of fibre reinforced polymer components. Proc. Inst. Civ. Eng.-Constr. Mater..

[6] Yanes-Armas S., Keller T. Structural concept and design of a GFRP-polyurethane sandwich roof structure. Proceedings of the Eighth International Conference on Fibre-Reinforced Polymer (FRP) Composites in Civil Engineering.

[7] Wang J., Song Y., Wang W., Tu L. (2018). Evaluation of composite crashworthy device for pier protection against barge impact. Ocean Eng..

[8] Alagusundaramoorthy P., Reddy R.V.S. (2008). Testing and evaluation of GFRP composite deck panels. Ocean. Eng..

[9] Abdolpour H., Garzón-Roca J., Escusa G., Sena-Cruz J.M., Barros J.A., Valente I.B. (2016). Development of a composite prototype with GFRP profiles and sandwich panels used as a floor module of an emergency house. Compos. Struct..

[10] Ndukwe C.O., Ezurike B.O., Okpala P.C. (2021). Comparative studies of experimental and numerical evaluation of tensile properties of Glass Fibre Reinforced Polyester (GFRP) matrix. Heliyon.

[11] Otoom O.F., Lokuge W., Karunasena W., Manalo A.C., Ozbakkaloglu T., Thambiratnam D. (2021). Experimental and numerical evaluation of the compression behaviour of GFRP-wrapped infill materials. Case Stud. Constr. Mater..

[12] Shekarchi M., Yekrangnia M., Biniaz A., Raftery G.M. (2021). Effect of elevated temperatures on the compressive behavior of timber filled steel and pultruded GFRP Beams. Compos. Struct..

[13] Al-Saadi A.U., Aravinthan T., Lokuge W. (2018). Structural applications of fibre reinforced polymer (FRP) composite tubes: A review of columns members. Compos. Struct..

[14] Zhang F., Liu W., Fang H., Xie G. (2018). Experimental study on flexural behavior of GFRP panel-cold-formed thin-walled steel composite beams. J. Build. Struct..

[15] Robinson M.J., Melby I.H. (2015). Effects of bonding in short-span rectangular concrete filled GFRP Beams. Compos. Struct..

[16] Abouzied A., Masmoudi R. (2017). Flexural behavior of rectangular FRP-Beams filled with reinforced concrete: Experimental and theoretical studies. Eng. Struct..

[17] Ahmed A.A., Hassan M., Masmoudi R. (2020). Effect of concrete strength and beams thickness on the flexural behavior of prestressed rectangular concrete-filled FRP Beams beams. Eng. Struct..

[18] Muttashar M., Manalo A., Karunasena W., Lokuge W. (2016). Influence of infill concrete strength on the flexural behaviour of pultruded GFRP square beams. Compos. Struct..

[19] Huang P., Yu H., Feng J., Hao G., Li Z. (2022). Experimental study on flexural behavior of GFRP profile-wood composite beam. J. Hebei Univ. Sci. Technol..

[20] Yoganantham C., Joanna P. (2021). Flexural behaviour of pultruded GFRP beams infilled with HVFA ECC. Mater. Today Proc..

[21] Zhang F., Lu Z., Wang D., Fang H. (2024). Mechanical properties of the composite sandwich structures with cold formed profiled steel plate and balsa wood core. Eng. Struct..

[22] Palomo I.R.I., Frappa G., de Almeida L.C., Trautwein L.M., Pauletta M. (2024). Analytical and numerical models to determine the strength of RC exterior beam–column joints retrofitted with UHPFRC. Eng. Struct..

[23] Alyousef R., Abbass W., Aslam F., Shah M.I. (2023). Potential of waste woven polypropylene fiber and textile mesh for production of gypsum-based composite. Case Stud. Constr. Mater..

[24] Balti S., Boudenne A., Dammak L., Hamdi N. (2023). Mechanical and thermophysical characterization of gypsum composites reinforced by different wastes for green building applications. Constr. Build. Mater..

[25] Chen C., Fang L., Wang Y., Jiu S., Chen Y. (2023). Mechanical, thermal and microscopic properties of gypsum matrix composites containing capric acid-palmitic acid/urea-formaldehyde microcapsules. Case Stud. Constr. Mater..

[26] Ding X., Huang W., Li Y., Hu Z., Shan Z. (2023). Study on retarding feature and retardation mechanism of various retarding materials on gypsum as a construction material: A review. J. Build. Eng..

[27] Wu C., He J., Wang K., Yang L., Wang F. (2023). Enhance the mechanical and water resistance performance of flue gas desulfurization gypsum by quaternary phase. Constr. Build. Mater..

[28] Babu K.S., Ratnam C. (2021). Mechanical and thermophysical behavior of hemp fiber reinforced gypsum composites. Mater. Today Proc..

[29] Alcaraz J.S., Belda I.M., Sanchis E.J., Borrell J.M.G. (2019). Mechanical properties of plaster reinforced with yute fabrics. Compos. Part B Eng..

[30] Gou M., Zhao M., Zhou L., Zhao J., Hou W., Ma W., Hou Z. (2023). Hydration and mechanical properties of FGD gypsum-cement-mineral powder composites. J. Build. Eng..

[31] Ma X., Tan L., Lu Y., Yao W., Wei Y. (2023). Upcycling of waste plasterboard for the synthesis of high-quality gypsum-based 3D printing powder. Constr. Build. Mater..

[32] Zhu C., Zhang J., Peng J., Cao W., Liu J. (2018). Physical and mechanical properties of gypsum-based composites reinforced with PVA and PP fibers. Constr. Build. Mater..

[33] Kuqo A., Mai C. (2021). Mechanical properties of lightweight gypsum composites comprised of seagrass *Posidonia oceanica* and pine (*Pinus sylvestris*) wood fibers. Constr. Build. Mater..

[34] Li Z., Wang X., Yan W., Ding L., Liu J., Wu Z., Huang H. (2023). Physical and mechanical properties of gypsum-based composites reinforced with basalt, glass, and PVA fibers. J. Build. Eng..

[35] Elkin A., Konev S., Safonov A., Gusev S., Sergeichev I. (2024). Compressive residual strength of the pultruded glass-fiber composite after tension-compression fatigue. Compos. Part C Open Access.

[36] Guo R., Xian G., Li F., Li C., Hong B. (2021). Hygrothermal resistance of pultruded carbon, glass and carbon/glass hybrid fiber reinforced epoxy composites. Constr. Build. Mater..

[37] Shaowei L.U., Xie H., Chen P. (2008). Simulation and optimization of GFRP pultrusion process. J. Compos. Mater..

[38] (2019). Standard Test Method for Shear Properties of Composite Materials by the V-Notched Beam Method.

[39] (2007). Standard Test Method for Tensile Properties of Polymer Matrix Composite Materials.

[40] (2016). Standard Test Method for Compressive Properties of Polymer Matrix Composite Materials with Unsupported Gage Section by Shear Loading.

[41] (2018). Steel for Reinforced Concrete Part 2, Hot Rolled Ribbed Bars.

[42] (2019). Standard for Test Methods of Physical and Mechanical Properties of Concrete.

[43] (2005). Standard for Test Methods of Flexural Properties of Fiber-Reinforced Plastics.

[44] Lejia L. (2014). Theoretical Bifurcation and Numerical Simulation of Flexural Behavior of FRP-Concrete Composite Beams. Master’s Thesis.

